# Self-Assembled Amphiphilic Fluorinated Random Copolymers for the Encapsulation and Release of the Hydrophobic Combretastatin A-4 Drug

**DOI:** 10.3390/polym14040774

**Published:** 2022-02-16

**Authors:** Matteo Calosi, Elisa Guazzelli, Simona Braccini, Marco Lessi, Fabio Bellina, Giancarlo Galli, Elisa Martinelli

**Affiliations:** Dipartimento di Chimica e Chimica Industriale, Università di Pisa, 56124 Pisa, Italy; matteocalosi@gmail.com (M.C.); elisa.guazzelli@dcci.unipi.it (E.G.); simona.braccini@phd.unipi.it (S.B.); marco.lessi@unipi.it (M.L.); fabio.bellina@unipi.it (F.B.); elisa.martinelli@unipi.it (E.M.)

**Keywords:** amphiphilic polymer, self-assembly, Combretastatin A-4, drug encapsulation, drug release

## Abstract

Water-soluble amphiphilic random copolymers composed of tri(ethylene glycol) methacrylate (TEGMA) or poly(ethylene glycol) methyl ether methacrylate (PEGMA) and perfluorohexylethyl acrylate (FA) were synthesized by ARGET-ATRP, and their self-assembling and thermoresponsive behavior in water was studied by dynamic light scattering (DLS) and UV-vis spectroscopy. The copolymer ability to self-fold in single-chain nano-sized structures (unimer micelles) in aqueous solutions was exploited to encapsulate Combretastatin A-4 (CA-4), which is a very hydrophobic anticancer drug. The cloud point temperature (*T*_cp_) was found to linearly decrease with increasing drug concentration in the drug/copolymer system. Moreover, while CA-4 was preferentially incorporated into the unimer micelles of TEGMA-*ran*-FA, the drug was found to induce multi-chain, submicro-sized aggregation of PEGMA-*ran*-FA. Anyway, the encapsulation efficiency was very high (≥81%) for both copolymers. The drug release was evaluated in PBS aqueous solutions both below and above *T*_cp_ for TEGMA-*ran*-FA copolymer and below *T*_cp_, but at two different drug loadings, for PEGMA-*ran*-FA copolymer. In any case, the release kinetics presented similar profiles, characterized by linear trends up to ≈10–13 h and ≈7 h for TEGMA-*ran*-FA and PEGMA-*ran*-FA, respectively. Then, the release rate decreased, reaching a plateau. The release from TEGMA-*ran*-FA was moderately faster above *T*_cp_ than below *T*_cp_, suggesting that copolymer thermoresponsiveness increased the release rate, which occurred anyway by diffusion below *T*_cp_. Cytotoxicity tests were carried out on copolymer solutions in a wide concentration range (5–60 mg/mL) at 37 °C by using Balb/3T3 clone A31 cells. Interestingly, it was found that the concentration-dependent micro-sized aggregation of the amphiphilic random copolymers above *T*_cp_ caused a sort of “cellular asphyxiation” with a loss of cell viability clearly visible for TEGMA-*ran*-FA solutions (*T*_cp_ below 37 °C) with higher copolymer concentrations. On the other hand, cells in contact with the analogous PEGMA-*ran*-FA (*T*_cp_ above 37 °C) presented a very good viability (≥75%) with respect to the control at any given concentration.

## 1. Introduction

Single-chain polymer nanoparticles (SCNPs) are a class of materials, developed mainly in the last two decades, supplying exceptionally small nanoparticles in the sub-20 nm range. This is accomplished thanks to their capability of self-folding by intramolecular interactions within a single polymer chain rather than forming multi-chain nanoobjects by intermolecular interactions [1,2,3,4].

SCNPs can be prepared in multiple ways, with intramolecular interactions created by both covalent and non-covalent bonds, which may be reversible or non-reversible [5,6]. A subset of SCNPs capable of reversible self-folding through non-covalent interactions is constituted by polymers that can self-fold through solvent-induced hydrophobic interactions in an aqueous environment. The research in this field has mainly concentrated on amphiphilic neutral random copolymers, where at least one comonomer provides hydrophilic properties and at least one other comonomer, as well as the main polymeric backbone, provides hydrophobic properties [7,8,9]. Amphiphilic random copolymer (ARP) SCNPs have attracted attention because their mechanism of intramolecular self-assembly into unimer micelles resembles the self-folding of proteins. Moreover, these ARP SCNPs are primarily formed by individual copolymers without the need for additional synthetic or post-modification steps and represent the most straightforward SCNP synthesis [10,11].

Poly(ethylene glycol) methacrylate (PEGMA) has been the most studied hydrophilic comonomer for SCNPs formed by non-ionic amphiphilic random copolymers, including the first synthesis of this kind of structure, where the hydrophobic comonomer was a methacrylate with alkyl pendant groups [12]. A different kind of hydrophobic comonomer, a methacrylate that included perfluorinated alkyl pendants, was soon thereafter tested in copolymers with PEGMA by Koda et al., which showed the same capability of self-folding into compact unimer micelles [13]. These copolymers were also demonstrated to be capable of undergoing reverse self-folding onto hydrophilic cores when using a fluorinated solvent. Furthermore, the self-folded micelles possessed a lower critical solution temperature (LCST)-type transition in both water and fluoroalkane [14]. However, the incorporation of the fluorinated co-units in the amphiphilic copolymers led to a lower LCST than that reported for analogous non-fluorinated ones. The inclusion of low surface energy fluoroalkyl side chains in a polymer structure is, in fact, known to drastically enhance the hydrophobic and lipophobic nature of the entire system, which results in a strong tendency to self-assemble in solution, in bulk and at the surface of thin films [15,16,17,18]. Follow-up studies by our group on similar copolymers of PEGMA and perfluorohexylethyl acrylate (FA) identified the formation of self-folded nanostructures by several complementary experimental analyses, including neutron scattering and molecular dynamics simulations, and we also studied the thermoresponsive features of the copolymers, which reversibly collapsed and aggregated into multi-chain aggregates at temperatures varying with copolymer composition [19,20,21,22].

A potential field of application of SCNPs that has seen increasing interest is their use as drug delivery systems [11,23,24,25]. The size of SCNPs (generally <10 nm in diameter) is significantly smaller than that of conventional polymer nanocarriers and more akin to that of globular proteins or small viruses. The study of how this may affect the bioavailability of the drug encapsulated in SCNP carriers is still in the early stages but points to their increased cellular uptake compared to larger nanoparticles [26,27,28,29,30]. The successful encapsulation of small hydrophobic molecules has been carried out in covalently crosslinked SCNPs [26,31,32] as well as in self-folding amphiphilic SCNPs [33,34]. As examples of this latter category, SCNPs obtained by grafting poly(vinyl acetate) on poly(ethylene glycol) were shown to be able to encapsulate a range of poorly water-soluble flavor molecules [34]. The encapsulation of a fluorinated agrochemical was also achieved, using random copolymers of PEGMA, a perfluoroalkyl methacrylate, and trehalose methacrylate. However, the presence of the agrochemical was found to promote the self-assembly of the copolymers into multi-chain structures rather than unimer micelles, with the pesticide acting as a physical fluorous crosslinker [35].

Many of the nanoobjects obtained from the self-assembling of amphiphilic copolymers containing PEG side chains possess an LCST in aqueous solution, which can be tuned by adjusting the copolymer composition [14,22,36]. Thermoresponsive nanoscale polymeric systems possessing an LCST have seen extensive study during the last two decades as potential drug carriers. These systems may be capable of releasing a substance at a much faster rate when a given temperature is reached due to the structural changes caused by the phase transition, as may be the case for example in tumoral tissues or by the induced hyperthermia of certain body areas [37]. By comparison to other nanoscale polymeric systems, SCNPs have seen little study as potential thermoresponsive drug release systems. An attempt has been made to exploit this property in a copolymer of PEGMA and a monomer containing uracil-diamido-pyridine, which formed SCNPs through hydrogen bonding [38]. This polymer was shown to be capable of thermo- and pH-responsive release behavior for the hydrophilic chemotherapy drug 5-fluorouracil. However, the drug promoted the assembly of the polymeric chains into larger multi-chain structures.

**Figure 1:**
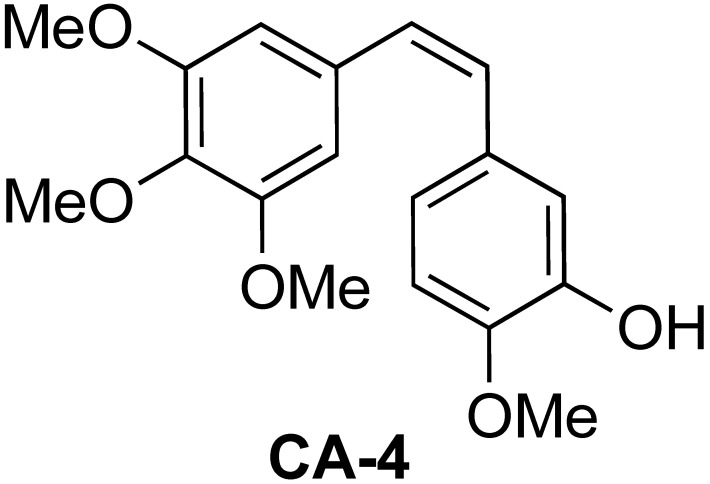
Chemical structure of *cis* Combretastatin A-4 (CA-4).

A potential drug molecule of interest for encapsulation in SCNPs is Combretastatin A-4 (CA-4) (Figure 1), which is the most active member of the Combretastatin A family isolated from the African tree *Combretum caffrum* [39].

CA-4 strongly inhibits tubulin assembly by binding to the colchicine binding site on tubulin [40,41,42]. Importantly, CA-4 displays a potent vascular disrupting activity, selectively damaging the tumor vasculature at doses that are substantially lower than those required to cause cytotoxicity [43,44,45,46,47,48]. The cascade of events that starts from the modification of microtubule cytoskeleton leads, ultimately, to rapid vasculature collapse and central tumor necrosis. However, a limitation to the clinical development of CA-4 has been its poor solubility in water and its low half-life in blood. CA-4 has been successfully encapsulated in polymeric systems, including liposomes [49], core–shell conventional polymeric nanoparticles [50,51], hydrogels [52]^,^ and cyclodextrin-based amphiphiles [53]. Its encapsulation in structurally simpler amphiphilic random copolymers able to self-assemble in smaller nanoobjects in water solution may represent a novel promising approach to overcome its inadequate solubility and improve stability, bioavailability, and efficacy.

In this work, we encapsulated CA-4 in two different amphiphilic random copolymers capable of self-folding into unimer micelles [20,22], composed, in one case, of tri(ethylene glycol) methyl ether methacrylate (TEGMA) and FA (TEGMA-*ran*-FA) and in the other case of PEGMA and FA (PEGMA-*ran*-FA). Both copolymers were found to display an LCST-behavior, with a cloud point temperature of 37 °C for TEGMA-*ran*-FA and well above the body temperature for PEGMA-*ran*-FA. We studied the effect of the drug on the self-folding, self-assembly, and thermoresponsive behavior of the copolymers in aqueous solution as well as the kinetics of drug release of CA-4 at temperatures below and above the cloud point temperatures. This enabled investigation of the role of thermoresponsiveness on the drug release in the latter case.

## 2. Results and Discussion

### 2.1. Syntheses of the Copolymers

**Figure 2:**
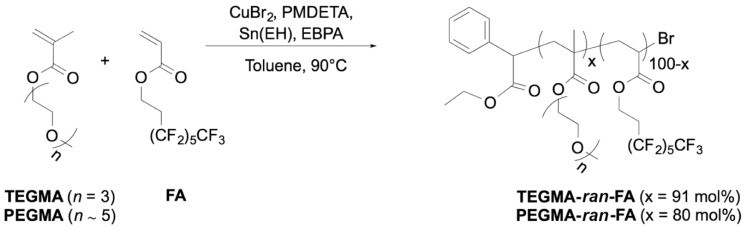
Schematic of the synthesis of the amphiphilic random copolymers via ARGET-ATRP.

Two different amphiphilic random copolymers were synthesized by activators regenerated by electron transfer atom transfer radical polymerization (ARGET-ATRP) of hydrophobic perfluorohexylethyl acrylate (FA) with a hydrophilic oligo(ethylene glycol) methyl ether methacrylate containing either three repeating ethylene glycol units (TEGMA) or five repeating units on average (PEGMA) (Figure 2). Different lengths of oligo(ethylene glycol) side chains were used to modulate the amphiphilicity of the copolymers, which was anticipated to affect the thermoresponsive behavior of the copolymer in water.

The ARGET-ATRP polymerization was carried out by using EBPA as initiator, CuBr_2_ as catalyst, PMDETA as ligand, Sn(EH)_2_ as reducing agent, and toluene as solvent at 90 °C, operating under vacuum. The EBPA/Sn(EH)_2_/CuBr_2_ molar ratio was 1/0.1/0.03. The selected synthetic conditions and comonomer feeds were tailored to obtain water-soluble copolymers with similar molecular weights, whose cloud point temperature would be around the human body temperature for TEGMA-*ran*-FA and above such temperature for PEGMA-*ran*-FA. The chosen reaction conditions and the properties of the obtained copolymers are shown in Table 1. The formation of the copolymers was confirmed by ^19^F NMR spectroscopy, which proved the insertion of the perfluorohexyl co-units in the polymer structure. The relative TEGMA or PEGMA and FA content was evaluated from the integrated areas of the ^1^H NMR characteristic signals (4.0–4.5 for O=COCH_2_ of both TEGMA (or PEGMA) and FA and 3.35 for OCH_3_ of TEGMA (or PEGMA)). Typically, the polymerizations yielded copolymers with comparable molecular weights (17–18 kg/mol) and narrow dispersities (*Đ* = 1.2–1.3).

### 2.2. Self-Assembling Behavior of Amphiphilic Copolymers

**Figure 3:**
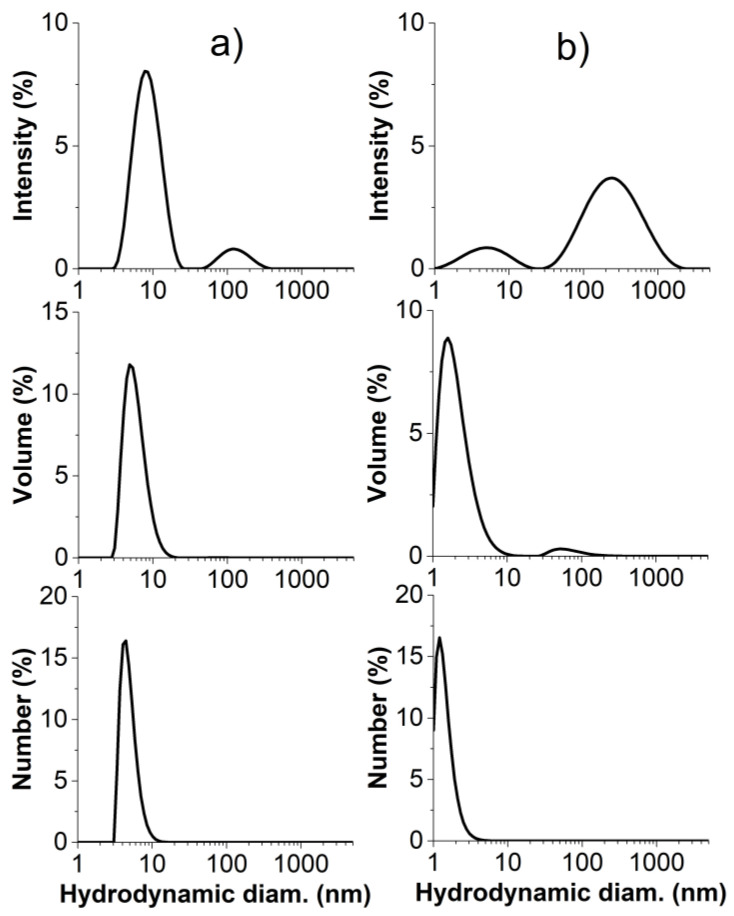
Intensity (**top**), volume (**middle**) and number (**bottom**) size distributions of (**a**) TEGMA-*ran*-FA and (**b**) PEGMA-*ran*-FA (25 °C, concentration of 30 g/L).

Copolymer water solutions were analyzed by dynamic light scattering (DLS) to determine their ability to spontaneously assemble in nanostructures at room temperature. As shown in Table 2, both copolymers had a bimodal intensity size distribution, with a population with a smaller hydrodynamic diameter *D*_h_ of 6–8 nm and a population with a much larger *D*_h_ of 120–400 nm. This is consistent with what was previously reported for related PEGMA-based fluorinated copolymers [13,19,20,22]. The smaller nanostructures were formed by the self-folding of single macromolecules into unimer micelles. Our investigations on such nanostructures by confirmatory small angle X-ray scattering (SAXS) measurements will be published in a forthcoming paper. A recently appeared small-angle neutron scattering (SANS) study carried out on similar PEGMA-*ran*-FA copolymers revealed that unimer micelles were compact globular, with radius of gyration *R*_g_ in the range 1.7−2.6 nm [22]. Such unimer micelles exhibited a core–shell nanostructure where hydrophilic oligoethylene glycol chains created a hydrated shell, shielding the compact hydrophobic core from the outer aqueous environment. This is in agreement with the analysis of folding trajectory and solvent-accessible surface area predicted by molecular dynamics (MD) simulations of such amphiphilic copolymers [20]. The population at smaller *D*_h_ was by far the dominant one in volume size distribution and the only one present in number size distribution (Figure 3), indicating that only a very minor fraction of the polymer chains was involved in the formation of larger size multi-chain aggregates induced by the intermolecular interactions between the least affine FA side chains with the water.

**Figure 4:**
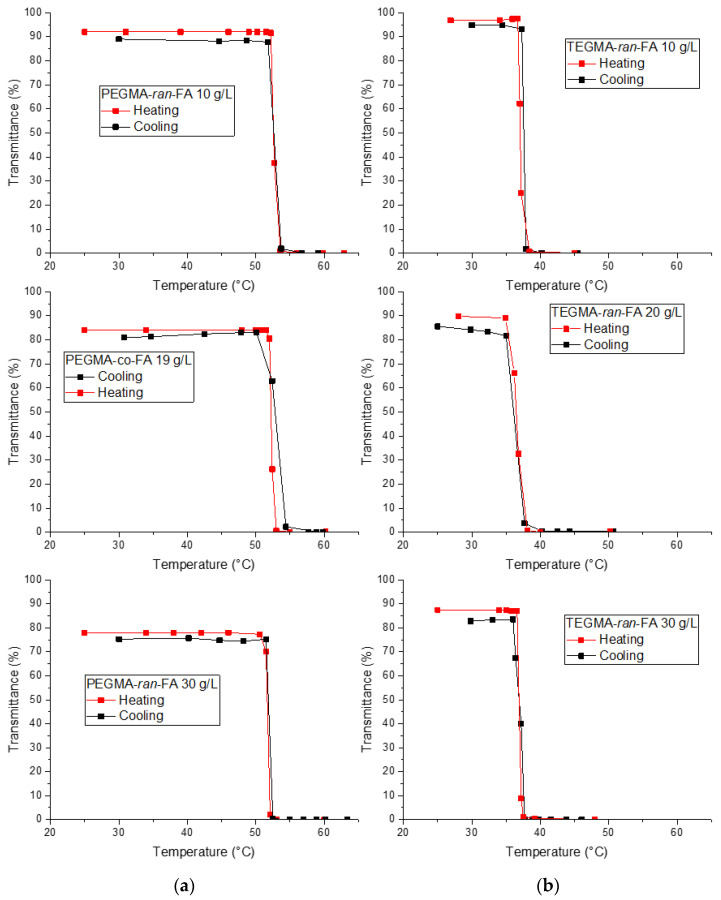
Turbidimetry measurements (λ = 700 nm) in water of PEGMA-*ran*-FA (**a**) and TEGMA-*ran*-FA (**b**) at varying concentrations.

Cloud point temperatures (*T*_cp_) of the copolymers in aqueous solutions were detected by turbidimetry as the temperatures at which light transmittance at 700 nm decreased to 50% of the original value, in order to identify the LCST-type behavior. The phase transition was found to be in any case sharp with no significant temperature hysteresis on cooling (Figure 4). The *T*_cp_ of TEGMA-*ran*-FA was located at 37 °C at 10 mg/mL and higher concentrations (Figure 4a). The *T*_cp_ of PEGMA-*ran*-FA was instead located at 52 °C at 10 mg/mL and higher concentrations (Figure 4b). Thus, TEGMA-*ran*-FA possessed thermoresponsive properties in the interval of human body temperature, while PEGMA-*ran*-FA only underwent the transition at higher temperatures. The higher *T*_cp_ of PEGMA-*ran*-FA for a given copolymer concentration was due to the higher hydrophilicity of the longer oxyethylenic side chains.

**Figure 5:**
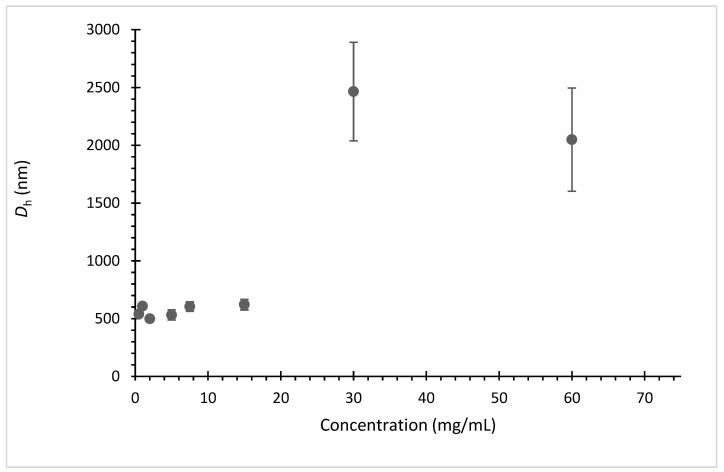
DLS hydrodynamic diameter (*D*_h_) of TEGMA-*ran*-FA aggregates in water at temperatures 5 °C above the respective *T*_cp_s.

The LCST-type transition resulted in a milky dispersion caused by the intermolecular aggregation of the polymer chains into larger particles. The nature of these aggregates was studied in more detail for TEGMA-*ran*-FA, presenting the *T*_cp_ around the human body temperature. TEGMA-*ran*-FA aggregates had a diameter of 400–600 nm, with low polydispersity, up to 15 mg/mL concentrations. Aggregates with much larger diameter, above 2000 nm with a high polydispersity, were formed when concentration was raised above this value (Figure 5). This was probably caused by further aggregation and precipitation when polymer concentration increased beyond the ≈15 mg/mL threshold. Our SANS studies on analogous PEGMA-*ran*-FA copolymers confirmed that compact, globular unimer micelles evolved into multi-chain aggregates having a rod-like shape and an *R*_g_ of 590–680 nm above *T*_cp_ [22].

### 2.3. Drug Encapsulation

**Figure 6:**
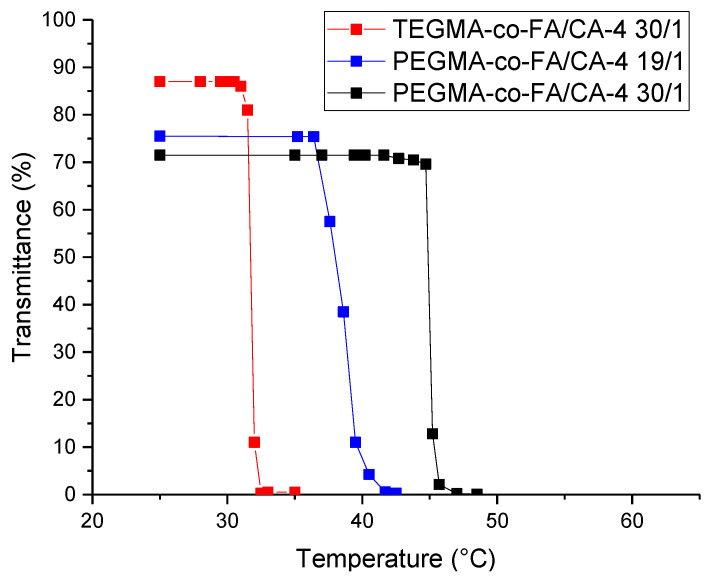
Turbidimetry measurements (λ = 700 nm) in water of polymer/drug systems TEGMA-*ran*-FA/CA4 30/1, PEGMA-*ran*-FA/CA4 30/1, and TEGMA-*ran*-FA/CA4 19/1.

The poorly water-soluble chemotherapy agent Combretastatin A-4 (CA-4) was tested as a model hydrophobic drug to be encapsulated within the unimer micelles derived from the copolymer single-chain folding in order to explore a potential application of the amphiphilic random copolymers. Drug encapsulation in the nanocarrier was achieved by a simple solvent evaporation method, wherein both CA-4 and the polymer were dissolved in a good organic solvent (dichloromethane), which was then removed under vacuum. This dry mixture of the two components was used as the polymer/drug formulation, to which the quantity of deionized water necessary to achieve a theoretical 1 mg/mL of CA-4 was added. Comparable results were also obtained when a PBS buffer solution was used. Owing to the self-folding of the amphiphilic random copolymers in unimer micelles, no further action other than stirring was needed to achieve encapsulation. Eventual amounts of unencapsulated drug were removed by centrifuging the solution at 500 rpm for 5 min, after which the supernatant was separated and analyzed for the CA-4 content by UV spectrophotometry. Various polymer/drug weight ratios were tested to find the optimal conditions for encapsulation. The lower ratios (≤10/1 for TEGMA-*ran*-FA and ≤5/1 for PEGMA-*ran*-FA) were found to result in very cloudy dispersions when water was added. This was caused by the decrease in the *T*_cp_ of the solution, from the one typical of the polymer alone in water to a value below room temperature. Higher polymer/drug weight ratios resulted in solutions transparent to the naked eye for TEGMA-*ran*-FA and weakly opaque solutions for PEGMA-*ran*-FA, being the light transmittance of the starting solutions 72–76% (Figure 6). For TEGMA-*ran*-FA, a 30/1 polymer/drug weight ratio was selected as the main formulation for the subsequent tests, as it was considered the lowest ratio at which *T*_cp_ (31–32 °C, Figure 6) was distant enough from room temperature to allow for measurements to be handily performed both above and below *T*_cp_. For PEGMA-*ran*-FA, tests were carried out at both polymer/drug weight ratios of 19/1 ratio (*T*_cp_ = 39 °C, Figure 6) and a 30/1 ratio (*T*_cp_ = 45 °C, Figure 6).

Very high encapsulation efficiency (Table 3, EE ≥ ≈81%) was obtained with both copolymers at all weight ratios, which was even larger where *T*_cp_ was kept above room temperature. Drug loading (DL) in the studied ratios ranged from 3 to 9%. Even though no inherent limitations were found for achieving higher values, higher loadings were not preferred in such a way to avoid resulting in *T*_cp_ values below room temperature. In terms of drug concentration, the obtained formulations were in the 800–900 ppm range. These values are more than one order of magnitude larger compared to normal CA-4 solubility in water (72 ± 3 ppm).

**Figure 7:**
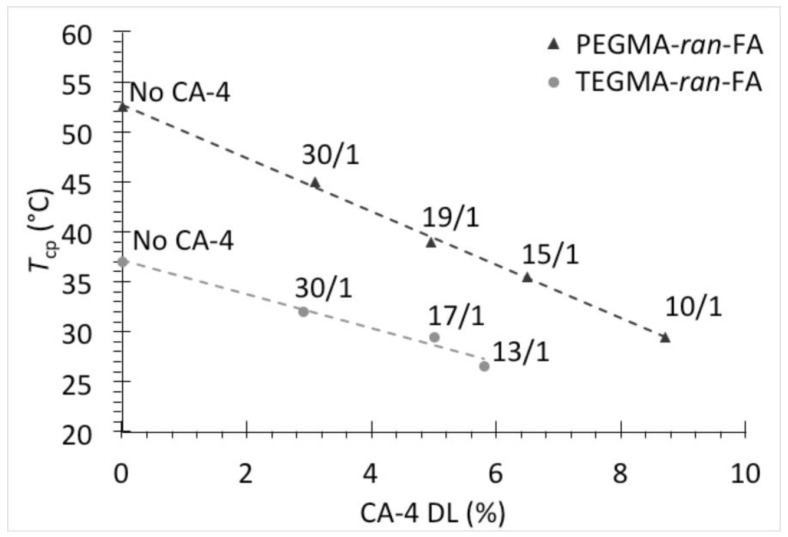
Cloud point temperature (*T*_cp_) of the systems PEGMA-*ran*-FA/CA-4 (triangles) and TEGMA-*ran*-FA/CA-4 (circles) as a function of drug loading (DL).

*T*_cp_ was found to decrease after the addition of the drug. This was due to the hydrophobic nature of CA-4 that preferentially interacted with the hydrophobic portions of the copolymer, thus leading to an overall increase in the hydrophobic/hydrophilic balance of the polymer/drug system, which in turn resulted in a reduction in *T*_cp_. Interestingly, *T*_cp_ values were found to linearly decrease by increasing the drug loading for both copolymer systems (Figure 7). Moreover, the *T*_cp_s of PEGMA-*ran*-FA/CA-4 systems were higher than those of the respective TEGMA-*ran*-FA/CA-4 ones, which is consistent with what was found for the copolymers alone.

The effect of the addition of a hydrophobic substance on the thermoresponsive behavior of amphiphilic random copolymers has, to our knowledge, not been investigated before. In this regard, a limited number of studies have been carried out on other systems capable of forming SCNPs. The effect we observed is analogous with the results obtained by Bartolini et al. for poly(vinyl acetate) grafted on poly(ethylene glycol), in which case a depression in *T*_cp_ by about 15 °C was noticed when anisaldehyde was encapsulated in a 1/1 weight ratio, although the more hydrophobic limonene had a much reduced effect on *T*_cp_ [34]. Cheng et al. [38] demonstrated that the encapsulation of fluorouracil into a PEGMA-based polymer modified with functional groups and capable of forming hydrogen bonds to obtain SCNPs resulted in a 20 °C decrease in *T*_cp_ [38]. However, in both cited examples, far higher weight ratios were needed to cause a change in *T*_cp_ on a similar scale to that of the copolymers in the present work.

**Figure 8:**
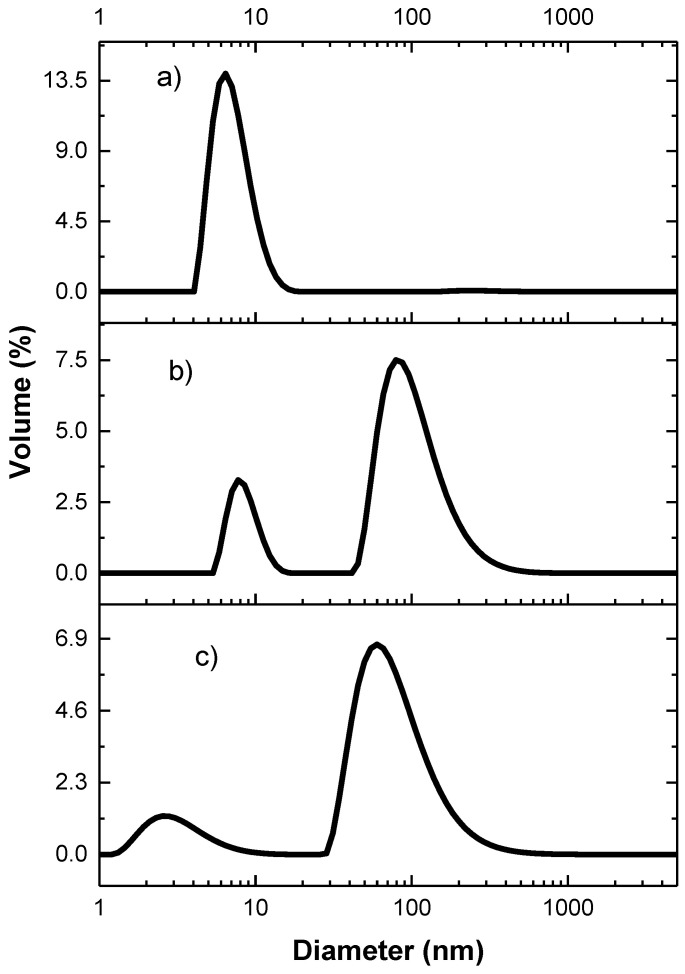
DLS volume size distribution at room temperature of TEGMA-*ran*-FA encapsulating CA-4 in a 30/1 weight ratio (**a**), PEGMA-*ran*-FA encapsulating CA-4 in 19/1 (**b**), and 30/1 weight ratios (**c**).

The aqueous dispersions of drug-loaded micelles were analyzed by DLS at room temperature to determine the effect of CA-4 on the self-assembling behavior of the copolymers. The size distribution was very similar to the one measured for the polymer itself (Figure 8a) when TEGMA-*ran*-FA was used in a 30/1 weight ratio, with the dominant peak in volume distribution being the one attributed to the self-folded nanostructure at 8 nm of diameter. Thus, the drug was predominantly encapsulated within the unimer micelles without causing a significant swelling of the micelle structure. By contrast, the dominant peak in both intensity and volume size distributions for the drug loaded micelles of PEGMA-*ran*-FA was located at higher values (Figure 8b,c), i.e., 530 for the 19/1 ratio and 320 for the 30/1 ratio systems in intensity size distribution. This suggests that in the latter case, the hydrophobic CA-4 acted as a physical crosslinker, inducing intermacromolecular interaction and aggregation into multi-chain structures. The different copolymer self-assembly behavior in the presence of the drug might be due to the lower stability of the PEGMA-*ran*-FA unimer micelles in aqueous solution with respect to the TEGMA-*ran*-FA counterparts because of the higher hydrophilicity of PEGMA. However, also in the PEGMA-*ran*-FA/CA-4 system, the unimer DLS component was still present as a minor population. Accordingly, this process was probably not a full transition but rather an equilibrium between the two different types of assemblies, with multi-chain structures being prevalent (Figure 8b,c). A difference between the two kinds of encapsulation was further proved by filtering the micelle dispersions through a 200 nm filter and analyzing the CA-4 content in the filtered sample. For PEGMA-*ran*-FA in a 19/1 weight ratio, this led to a 75 ± 9% decrease in CA-4 concentration, while for TEGMA-*ran*-FA, this decrease was 13 ± 7%. In a previous experiment carried out by Ko et al. with a similar random copolymer of PEGMA and FA, a heavily fluorinated small molecule was shown to instead cause the complete aggregation of the polymer into a unimodal population with a diameter of 340 nm [35]. Thus, the chemical nature of the encapsulated molecule played a decisive role in driving the amphiphilic random copolymer self-assembly, with the fluorinated molecule being able to promote multi-chain assembly by linking the fluorous side chains together, while a molecule with a lesser affinity for the fluorinated component such as CA-4 was capable of forming weaker nonspecific hydrophobic interactions with the fluorinated side chains.

**Figure 9:**
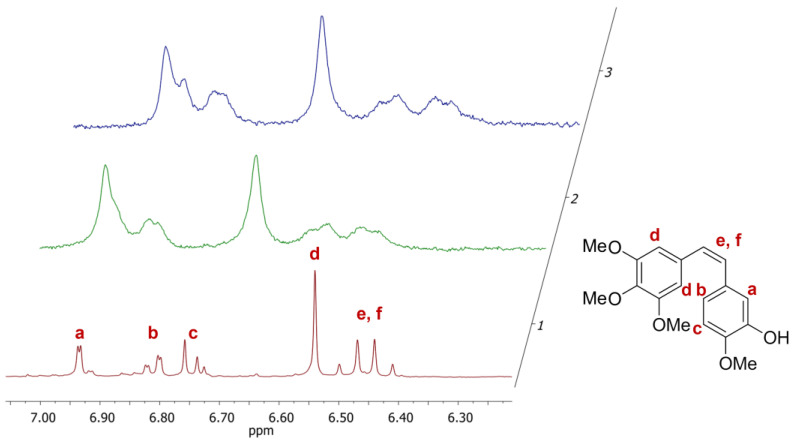
^1^H NMR spectra for the aromatic and double bond regions of CA-4 in CDCl_3_ (1), CA-4 encapsulated in TEGMA-*ran*-FA (2), and PEGMA-*ran*-FA (3) in D_2_O.

To gain more insight into the CA-4/polymer interactions, the polymer/drug systems were investigated by ^1^H NMR in D_2_O. The signals characteristic of CA-4 encapsulated in both TEGMA-*ran*-FA and PEGMA-*ran*-FA were significantly broader with respect to those typical of CA-4 in CDCl_3_ but still clearly visible (Figure 9). This indicates that the drug possessed a certain degree of mobility within the polymer micelles, differently from what was reported for other hydrophobic drugs whose signals completely disappeared due to lack of mobility once encapsulated within polymer core–shell nanoparticles [54,55,56,57].

### 2.4. Drug Release

**Figure 10:**
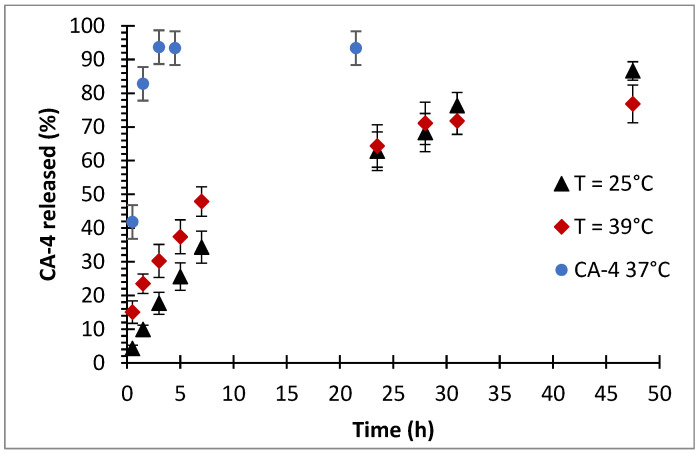
Release profiles of CA-4 from TEGMA-*ran*-FA with a 30/1 polymer/drug weight ratio at temperatures below and above *T*_cp_. As a reference, the release profile of CA-4 from a 1 g/L solution in DMSO is also shown.

**Figure 11:**
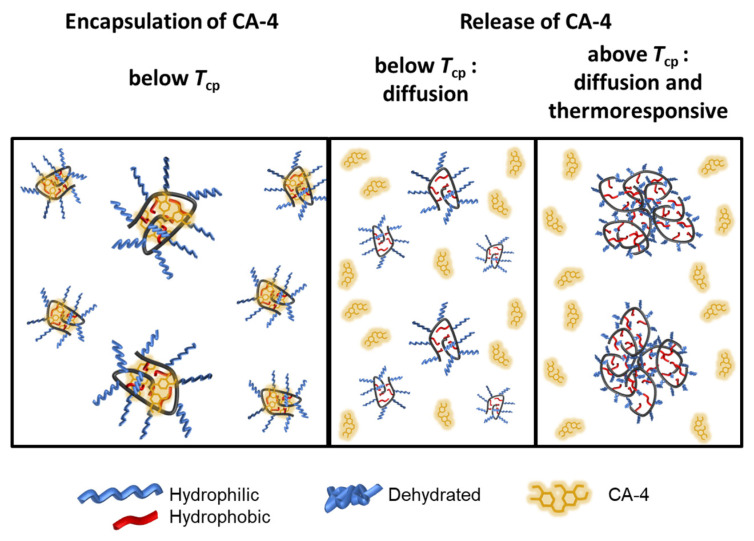
Schematic representation of the mechanism of release of the encapsulated CA-4 drug from TEGMA-*ran*-FA unimer micelles, below and above *T*_cp_.

CA-4 release profiles for both copolymers were recorded using a dialysis method, with phosphate-buffered saline (PBS, pH = 7.4) serving as the release medium. We also recorded a profile of the free diffusion of CA-4 from inside the dialysis tubing to demonstrate the actual establishment of sink condition, as can be seen in Figure 10. TEGMA-*ran*-FA showed at room temperature a sustained release profile, resulting in 60% drug release in 24 h and 85% release in 48 h (Figure 10). CA-4 retained a certain mobility while encapsulated in the core instead of being strongly confined inside it and could diffuse away when in sink conditions as a result. Encapsulation did slow this process down significantly, as almost 90% release took two days to be achieved instead of 3 h. At 39 °C, TEGMA-*ran*-FA showed a higher initial release compared to that at room temperature (15% vs. 5% drug release in the initial 30 min), after which it gradually trended to the same cumulative release, reaching equal values after 24 h and retaining a slightly higher quantity of CA-4 after 48 h (Figure 10). Thus, the results indicate that the drug release occurred, in any case, by diffusion at temperature below *T*_cp_, and thermoresponsiveness was not a necessary condition to trigger the release. However, the latter seemed to speed up the release in the initial linear region up to 10 h, suggesting that above *T*_cp_, release occurred by a combination of diffusion and thermoresponsive mechanisms rather than with a fully thermoresponsive release as was reported for other polymer/drug systems [37,58,59]. In particular, above *T*_cp_, the stable nanoassemblies containing the drug collapsed into much larger multi-chain aggregates that expelled solvation water molecules no longer engaged in hydrogen bonds with the oxyethylene side chains and simultaneously released the drug to the outer environment (Figure 11). This is consistent with what was previously found for a similar PEGMA-*ran-*FA amphiphilic copolymer embedding ethidium bromide as a fluorescent probe [20].

**Figure 12:**
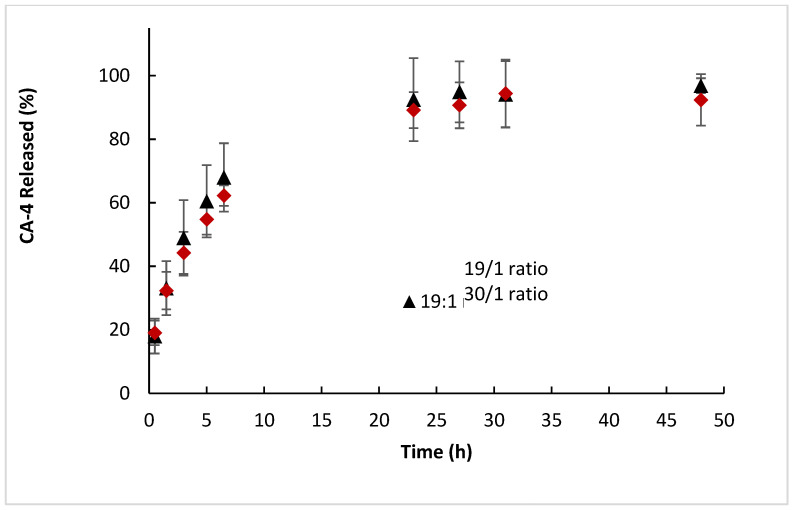
Release profiles of CA-4 from PEGMA-*ran*-FA, using different polymer/drug weight ratios, at 37 °C.

Release profiles of PEGMA-*ran*-FA were recorded using both 19/1 and 30/1 weight ratio systems while operating at the human body temperature of 37 °C, i.e., below *T*_cp_ for both formulations (Figure 12). Both systems resulted in very similar release profiles, indicating that the drug release was not significantly affected by copolymer concentration or the difference between the *T*_cp_s and the actual temperature of the test.

Overall, PEGMA-*ran*-FA showed a significantly faster drug release behavior compared to TEGMA-*ran*-FA, even when the latter was recorded above its *T*_cp_, with around half the encapsulated drug being released in 5 h and near-full release (85–90%) within a day from the beginning of the experiment. Thus, CA-4 seemed to retain a greater mobility within the multi-chain aggregates compared to that when it was encapsulated inside the unimer micelles of TEGMA-*ran*-FA. However, the drug release was also in this case significantly lower than for the free, i.e., non-encapsulated, system.

### 2.5. Cytotoxicity Assay

The cytocompatibility of copolymers PEGMA-*ran*-FA and TEGMA-*ran*-FA toward the Balb/3T3 clone A31 cell line was assessed by directly incubating the cells in copolymer solutions with different concentrations (5–60 mg/mL). In WST-1 assay, only cells that were viable after 24 h of exposure to the sample were capable of efficiently metabolizing the dye (4-[3-(4-iodophenyl)-2-(4-nitro-phenyl)-2H-5-tetrazolio]-1,3-benzene sulfonate) to formazan, which was subsequently analyzed spectrophotometrically.

**Figure 13:**
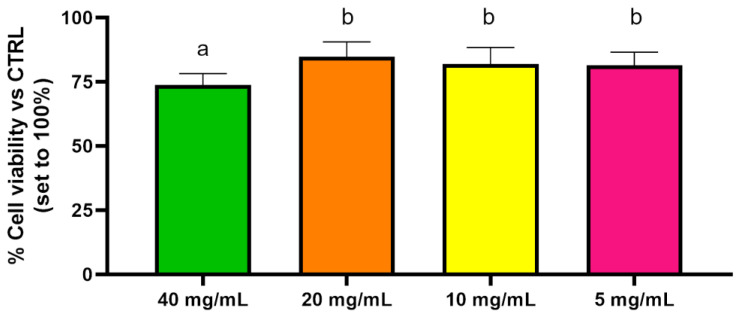
Cell viability evaluation of Balb/3T3 clone A31 cultured with several concentrations of PEGMA-*ran*-FA for 24 h. Not-shared letters indicate statistically significant differences, *p* < 0.05.

**Figure 14:**
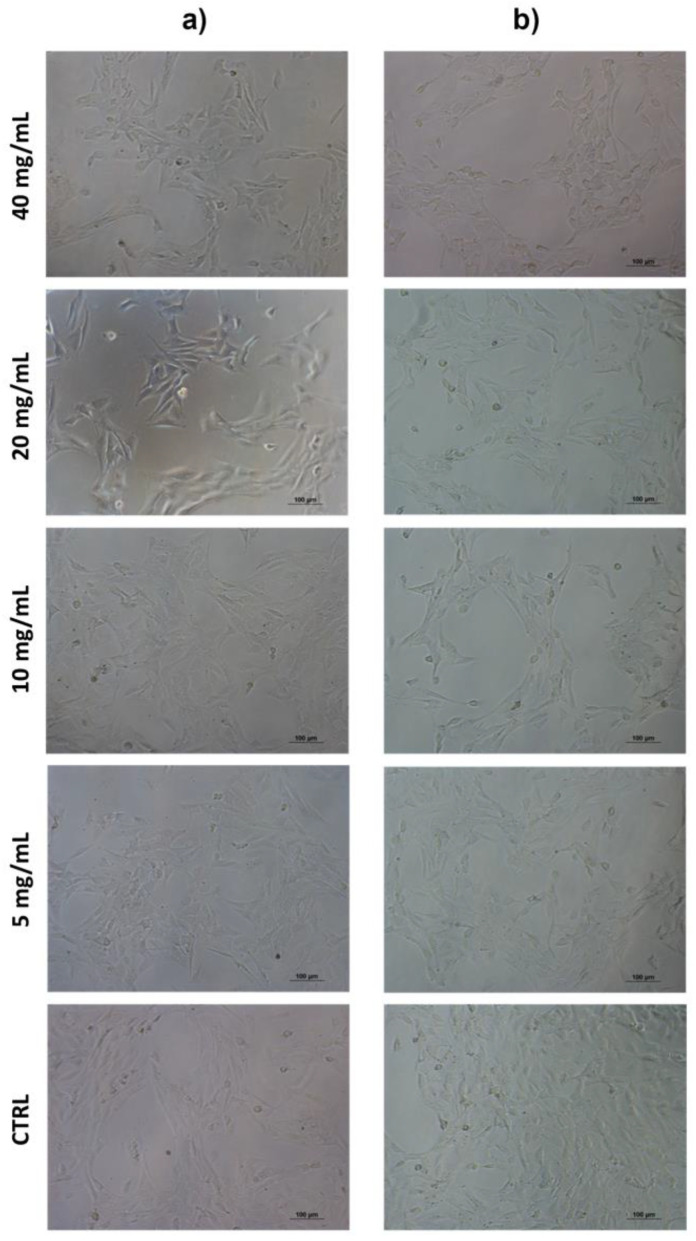
Optical micrographs of Balb/3T3 clone A31 cultured with several concentrations of PEGMA-*ran*-FA for 24 h (10× magnification). Photos taken after cell incubation at 37 °C (**a**) and after washing at room temperature (**b**).

**Figure 15:**
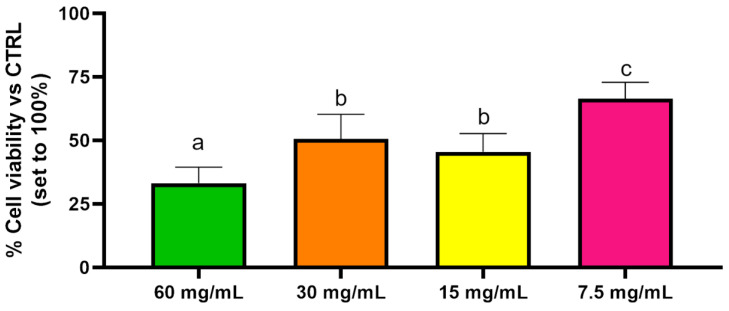
Cell viability evaluation of Balb/3T3 clone A31 cultured with several concentrations of TEGMA-*ran*-FA for 24 h. Not-shared letters indicate statistically significant differences, *p* < 0.05.

**Figure 16:**
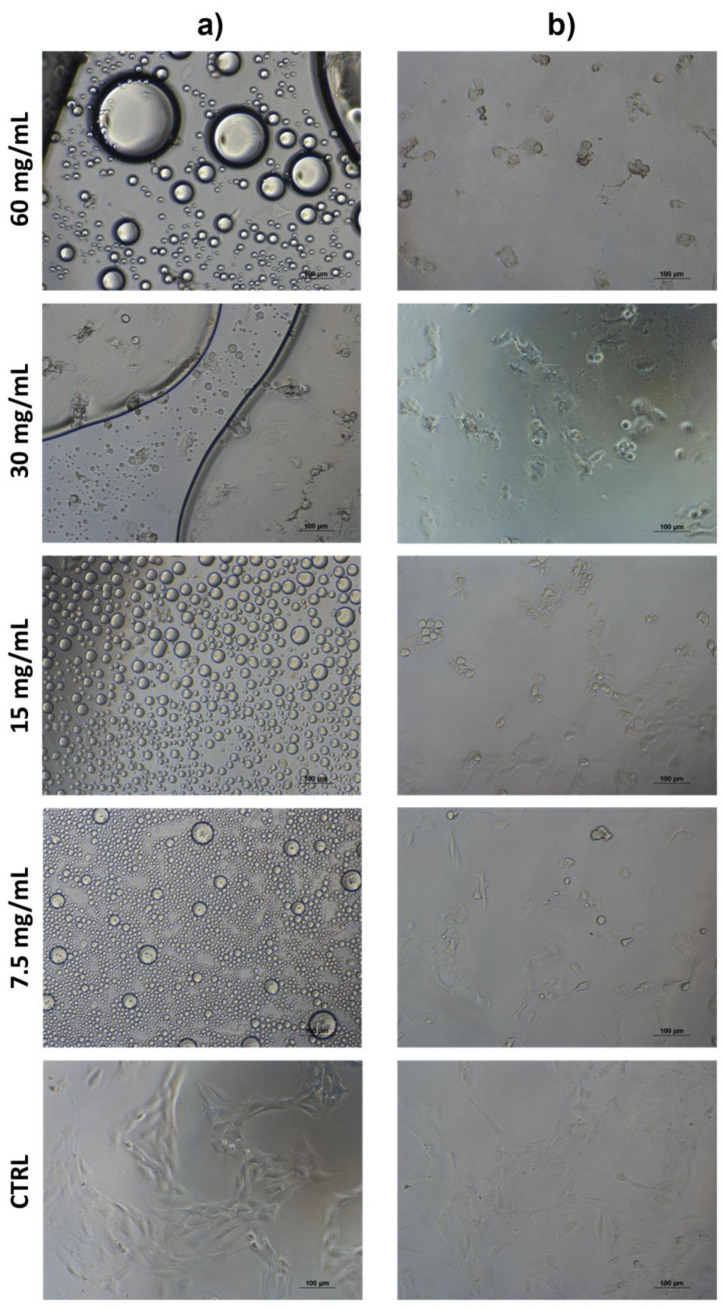
Optical micrographs of Balb/3T3 clone A31 cultured with several concentrations of TEGMA-*ran*-FA for 24 h (10× magnification). Photos taken after cell incubation at 37 °C (**a**) and after washing at room temperature (**b**).

The results showed that Balb/3T3 clone A31 cells treated with PEGMA-*ran*-FA for 24 h displayed very good viability (≥75%) with respect to the control even at the maximum investigated copolymer concentration of 40 mg/mL (Figure 13) and a morphology typical of fibroblast (Figure 14). These findings confirmed the lack of toxicity of the copolymer toward the cell line. Toxicity effects of any residual traces of Cu-catalyst could also be ruled out. On the other hand, TEGMA-*ran*-FA exhibited an unexpectedly reduced cell viability (Figure 15) that tended to decrease by increasing copolymer concentration reaching the minimum level of 30% at 60 mg/mL. Such a concentration-dependent toxicity of TEGMA-*ran*-FA with respect to the non-toxic and structurally similar PEGMA-*ran*-FA was mostly likely due to the formation of multi-chain aggregates at 37 °C (> *T*_cp_) rather than to the inherent toxicity of the chemical constituents. As shown above (Figure 3), such aggregates presented concentration-dependent sizes that varied from ≈600 nm up to ≈2500 nm diameters in passing from 7.5 to 60 mg/mL, which are expected to completely cover the cellular monolayer, thus causing cell death. This is attributed to a sort of “cellular asphyxiation” due to the prevention of the normal acquisition of nutrients and gases and the release of waste products [60]. As shown in Figure 16B, the cells after 24 h of incubation with TEGMA-*ran*-FA showed a reduced proliferation rate and a rounded shape, suggesting cellular suffering. On the contrary, cells assumed a stellate/elongated shape typical of fibroblasts on the control.

## 3. Materials and Methods

### 3.1. Polymer Synthesis

#### 3.1.1. Materials

Polyethyleneglycol methyl ether methacrylate (PEGMA, *M*_n_ = 300 g/mol, average degree of polymerization ≈5) (Sigma-Aldrich, Darmstadt, Germany), triethyleneglycol methyl ether methacrylate (TEGMA *M_n_* = 232 g/mol) (Sigma-Aldrich, Darmstadt, Germany), and perfluorohexylethyl acrylate (FA, Fluorochem, Hadfield, United Kingdom) were passed through basic alumina to remove inhibitors. *N,N,N′,N″,N″*-pentamethyldiethylenetriamine (PMDETA, Sigma-Aldrich) and ethyl *α*-bromophenylacetate (EBPA, Sigma-Aldrich, Darmstadt, Germany) were distilled under vacuum. CuBr_2_ (Sigma-Aldrich, Darmstadt, Germany) was recrystallized from water solution. Toluene (Sigma-Aldrich, Darmstadt, Germany) was distilled at atmospheric pressure after reflux over calcium hydride. 3-Hydroxyanisaldehyde, 3,4,5-trimethoxyphenylacetic acid, acetic anhydride, triethyl amine, and copper (powder) were used as received (Sigma-Aldrich, Darmstadt, Germany). Quinoline was distilled under reduced pressure before use.

The preparation of the *cis*-Combretastatin A-4 (CA-4) was carried out following a two-step literature procedure, obtaining the desired product with a 98% GLC purity and a 40% yield [61].

Common laboratory solvents and other reagents (Sigma-Aldrich, Darmstadt, Germany) were used as received.

#### 3.1.2. Synthesis of Copolymer TEGMA-*ran*-FA

The CuBr_2_/PMDETA complex (0.0061 mmol) was first added as a 0.0134 M solution in methanol (3 g/L of CuBr_2_) in a 50 mL Carius tube, which was then dried under vacuum for 1 h. Then, TEGMA (2.58 mL, 11.4 mmol), FA (0.23 mL, 0.86 mmol), EBPA (35.8 µL, 0.20 mmol), and toluene (8 mL) were added to the tube, and the mixture was degassed with two freeze–pump–thaw cycles. Then, SnEH_2_ (16.6 mg, 0.041 mmol) and PMDETA (7.1 µL, 0.0041 mmol) in 0.4 mL of toluene were added, and three more freeze–pump–thaw cycles were performed before the polymerization was started at 90 °C, under vacuum and with magnetic stirring. After 27 h, the reaction was stopped by exposure to air and quenching to room temperature. The crude product was precipitated twice from chloroform solutions into *n*-hexane, then dissolved in chloroform, passed through a neutral alumina column until complete discoloration, and evaporated to dryness under vacuum.

^1^H NMR (acetone-d_6_): δ (ppm) = 7.1–7.4 (aromatic), 4.0–4.5 (O=COCH_2_), 3.5–3.8 (OCH_2_), 3.35 (OCH_3_), 2.5–2.7 (CH_2_CF_2_), 1.8–2.1 (CH_2_, CH), 0.8–1.1 (CH_3_).

^19^F NMR (acetone-d_6_, CF_3_COOH): δ (ppm) = –5.6 (CF_3_), –38.5 (CF_2_CH_2_), –46 to –48 (CF_2_), –51 (CF_2_CF_3_).

#### 3.1.3. Synthesis of Copolymer PEGMA-*ran*-FA

The CuBr_2_/PMDETA complex (0.0072 mmol) was first added as a 0.0134 M solution in methanol (3 g/L of CuBr_2_) in a 50 mL Carius tube, which was then dried under vacuum for 1 h. Then, PEGMA (2.76 mL, 9.67 mmol), FA (0.65 mL, 2.42 mmol), EBPA (42.3 µL, 0.24 mmol), and toluene (10 mL) were added to the tube, and the mixture was degassed with two freeze–pump–thaw cycles. Afterwards, SnEH_2_ (9.8 mg, 0.024 mmol) and PMDETA (5.0 µL, 0.024 mmol) in 0.2 mL of toluene were added and three more freeze–pump–thaw cycles were performed before the polymerization was started at 90 °C, under vacuum and with magnetic stirring. After 18 h, the reaction was stopped by exposure to air and quenching to room temperature. The crude product was precipitated twice from chloroform solutions into *n*-hexane, then dissolved in chloroform, passed through a neutral alumina column until complete discoloration, and evaporated to dryness under vacuum.

^1^H NMR (acetone-d_6_): δ (ppm) = 7.2–7.4 (aromatic), 4.0–4.5 (O=COCH_2_), 3.5–3.8 (OCH_2_), 3.35 (OCH_3_), 2.6–2.8 (CH_2_CF_2_), 1.8–2.1 (CH_2_, CH), 0.8–1.1 (CH_3_).

^19^F NMR (acetone-d_6_, CF_3_COOH): δ (ppm) = −5.6 (CF_3_), −38.5 (CF_2_CH_2_), −46 to −48 (CF_2_), −51 (CF_2_CF_3_).

### 3.2. Characterization

^1^H and ^19^F NMR measurements were carried out on a Bruker Avance 400 (400 MHz, Billerica, MA, USA) spectrometer with deuterated solvents at room temperature. The sample concentration was approximately 30 g/L. For ^19^F NMR chemical shift attribution, trifluoacetic acid was used as internal standard. For ^1^H spectra, the internal standard was the solvent peak.

Gas liquid chromatography analyses were performed on a Dani GC 1000 (Milano, Italy) instrument equipped with a PTV injector and recorded with a Dani DDS 1000 data station. Two types of capillary columns were used: an Agilent J&W DB-5 column (Folsom, CA, USA) (30 m × 0.25 mm i.d. × 0.25 μm) and an Agilent J&W DB-1 column (Folsom, CA, USA) (15 m × 0.25 mm i.d. × 0.25 μm). EI-MS spectra were recorded at 70 eV by GLC-MS, performed on an Agilent 6890N (Waldbronn, Germany) gas-chromatograph interfaced with an Agilent 5973N mass detector, using Agilent J&W DB-5MS column (Folsom, CA, USA) (30 m × 0.25 mm i.d. × 0.25 μm) as capillary column.

The number and weight average molecular weights (*M*_n_, *M*_w_) were determined by gel permeation chromatography (GPC), using a Jasco (Hachioji-shi, Tokyo, Japan) PU-2089 Plus liquid chromatograph equipped with two PL gel 5 µm mixed-D columns, a Jasco RI-2031 Plus refractive index detector, and a Jasco (Hachioji-shi, Tokyo, Japan) UV-2077 Plus UV/vis detector. Measurements were carried out using chloroform as the mobile phase, at a flux of 1 mL/min and a temperature of 30 °C maintained by a Jasco (Hachioji-shi, Tokyo, Japan) CO 2063 Plus column thermostat. Samples were filtered with a 0.2 µm PTFE filter before injection. Poly(methyl methacrylate) standards were used for calibration. The refractive index detector was used to obtain reported values.

Turbidimetry measurements were carried out using a Perkin-Elmer (Waltham, MA, USA) Lambda 650 spectrometer. Samples were put into quartz cuvettes with a 10 mm optical path. Light transmittance was measured at a fixed wavelength of 700 nm. The cloud point temperature (*T*_cp_) was defined as the temperature at which light transmittance decreased to 50% of the initial baseline. Aqueous solutions of the polymers at different concentrations (from 0.5 to 30 g/L) were tested. Measures were taken at various temperatures, ranging from room temperature to higher than *T*_cp_ of the given copolymer. Temperature was varied manually using a thermostat.

UV-Vis spectrophotometry measurements were carried out with a JASCO (Hachioji-shi, Tokyo, Japan) V-750 spectrometer using quartz cuvettes with a 10 mm optical path. Absorbance of the samples was compared to a calibration curve at the appropriate wavelength. A calibration curve for CA-4 in DMSO solution was obtained using concentrations of CA-4 between 2 and 80 ppm and measuring their absorbance at 310 nm. A calibration curve for CA-4 in water was obtained by diluting with deionized water a 1 g/L solution of CA-4 in ethanol over a concentration range between 0.2 and 20 ppm and measuring absorbance of the resulting samples at 295 nm.

Dynamic light scattering (DLS) measurements of polymer solutions were taken with a Beckman Coulter (Brea, CA, USA) Delsa Nano C particle analyzer (detection angle = 166.22°). The intensity, volume, and number distributions were obtained from the signal autocorrelation function through CONTIN analysis in the instrument software. Sample equilibration time was 60 s for measurements at room temperature and 300 s for measurements at higher temperatures. Samples were prepared in previously filtered water (0.2 µm cellulose acetate filters) to reduce external contamination. At least 5 separate measurements were carried out for each solution. Hydrodynamic diameters were always calculated according to the intensity size distribution and averaged between the various measurements. Reported graphs were from a single measurement taken as typical of the series.

### 3.3. Solubility Assay for Combretastatin A-4 in Water

First, 1 mL of deionized water was added to 1 mg of CA-4, and the resulting dispersion was magnetically stirred overnight, after which it was transferred in a 1.5 mL Eppendorf tube and centrifuged at 500 rpm for 5 min. Then, the supernatant solution was diluted with deionized water, and the absorbance of this solution was measured at 295 nm and compared to the reference curve for CA-4 in water.

### 3.4. Preparation of Combretastatin A-4 Loaded Micelles

The synthesized copolymer and CA-4 were dissolved in dichloromethane in the desired proportions, after which the obtained solutions were magnetically stirred overnight. Then, the organic solvent was removed under vacuum, and deionized water was added in the volume necessary to obtain a 1 g/L concentration of CA-4. The resulting dispersion was magnetically stirred overnight, after which it was transferred in 1.5 mL Eppendorf tubes and centrifuged at 500 rpm for 5 min. The supernatant solution was diluted with DMSO, and the absorbance of this solution was measured at 310 nm and compared to the reference curve for CA-4 in DMSO.

Drug loading (DL) and encapsulation efficiency (EE) of the micelles were obtained by Equations (1) and (2):

(1) $$\mathrm{DL}=\;\frac{\mathrm{Amount}\;\mathrm{of}\;\mathrm{drug}\;\mathrm{in}\;\mathrm{micelles}}{\mathrm{Amount}\;\mathrm{of}\;\mathrm{drug}+\mathrm{polymer}}\;\cdot 100\%$$

(2) $$\mathrm{EE}=\frac{\mathrm{Amount}\;\mathrm{of}\;\mathrm{drug}\;\mathrm{in}\;\mathrm{micelles}}{\mathrm{Amount}\;\mathrm{of}\;\mathrm{drug}\;\mathrm{in}\;\mathrm{the}\;\mathrm{feed}}\;\cdot 100\%.$$

The measured solubility of CA-4 was assumed to also be the maximum possible amount of unencapsulated drug present and thus subtracted from the amount of measured drug to obtain the minimum possible amount of drug in micelles.

### 3.5. In Vitro Drug Release

An aliquot of 0.7–1.2 mL of the micelle dispersion was placed in a dialysis bag (Spectrapor Biotech regenerated cellulose dialysis tubing, MWCO 3.5–5.0 kDa) and dialyzed against 300 mL of PBS (pH 7.4) in a 400 mL beaker. At set time intervals, 2.5 mL aliquot of the release medium was removed and replaced with the same volume of PBS. The absorbance of the samples was measured at 295 nm and compared to the reference curve for CA-4 in water. Percentage released at each time was calculated by comparison to the total drug concentration in the original formulation. Data are presented as averaged from three separate release tests. A diffusion test of CA-4 at 37 °C was also carried out in which 1 mL of a 1 mg/mL solution of CA-4 in DMSO was placed inside the dialysis tubing.

### 3.6. Cytotoxicity Assay

A mouse embryo fibroblast Balb/3T3 clone A31 cell line from the American Type Culture Collection (ATCC CCL-163) was selected to investigate the cytocompatibility of the amphiphilic polymers. Cells were propagated as indicated by the supplier using Dulbecco’s Modified Eagle Medium (DMEM) (Sigma-Aldrich, Darmstadt, Germany) supplemented with 4 mM of L-glutamine (Sigma-Aldrich, Darmstadt, Germany), 1% of penicillin:streptomycin solution (10,000 U/mL:10 mg/mL; Sigma-Aldrich, Darmstadt, Germany), 10% of calf serum (Sigma-Aldrich, Darmstadt, Germany), and antimycotic (complete growth medium).

Balb/3T3 clone A31 cells were seeded in 96-well culture plates at a concentration of 4 × 10^3^/well. After overnight incubation at 37 °C in a 5% CO_2_-enriched atmosphere, cells were exposed to different concentrations of the copolymers (5–60 mg/mL) for 24 h. Cells incubated with complete growth medium were used as control (CTRL). Viability was investigated by mean of WST-1 tetrazolium salt reagent (Sigma-Aldrich, Darmstadt, Germany). Briefly, cells were washed twice with complete growth medium to remove any traces of the tested compounds and then were incubated for 4 h with WST-1 reagent diluted 1:10, at 37 °C, and 5% CO_2_. Measurements of the formazan dye absorbance were carried out with a microplate reader (Biorad, Milan, Italy) at 450 nm and 655 nm as a reference wavelength. The in vitro biological tests were performed on triplicate for each sample. Experimental data were reported as mean ± standard deviation, and statistical differences were analyzed by using one-way analysis of variance (ANOVA) followed by a Tukey post hoc test. A p value < 0.05 was considered statistically significant.

Optical microscopy observation of cells was performed with a Nikon Eclipse TE 2000 inverted microscope equipped with epifluorescence lamp and Nikon D Eclipse C1 confocal system (Nikon, Tokyo, Japan). Optical micrographs were taken immediately after incubation at 37 °C and after washing the samples to remove the copolymer.

## 4. Conclusions

Well-defined amphiphilic random copolymers TEGMA-*ran*-FA and PEGMA-*ran*-FA were synthesized by controlled ARGET-ATRP, and their self-assembling ability in water was exploited to encapsulate Combretastatin A-4 (CA-4), a very active, but poorly water-soluble, anticancer drug. Both amphiphilic copolymers were confirmed to predominantly self-fold in single-chain nanostructures, i.e., unimer micelles, in aqueous solution with small hydrodynamic diameters (≤8 nm) that thermoreversibly self-associated in larger concentration-dependent size multi-chain aggregates. The more hydrophilic PEGMA-*ran*-FA copolymer presented higher cloud point temperatures (*T*_cp_s) at the given concentrations. Encapsulation of drug occurred with a high efficiency (≥81%), although it was based on different mechanisms for the two copolymers. In particular, the drug was found to be incorporated in the unimer micelles of TEGMA-*ran*-FA, whereas it was preferentially encapsulated into multi-chain aggregates of PEGMA-*ran*-FA, whose formation was induced by the hydrophobic drug itself. The addition of CA-4 affected the thermoresponsiveness of copolymers causing a significant reduction in the *T*_cp_ which linearly decreased by increasing the amount of drug with respect to the *T*_cp_ of copolymer alone. Release of drug was significantly slowed down with respect to the free, i.e., not encapsulated, drug and presented a linear region up to ≈10–13 h and ≈7 h release for TEGMA-*ran*-FA and PEGMA-*ran*-FA, respectively. Release occurred at temperature below *T*_cp_, indicating that thermoresponsiveness was not necessary_,_ to trigger the process, even though it appeared to moderately increase the release rate over the linear region.

Cytotoxicity tests carried out using Balb/3T3 clone A31 cells showed that the formation of micro-sized aggregates of TEGMA-*ran*-FA at 37 °C, i.e., above *T*_cp_, drastically reduced cell viability, causing a sort of “cellular asphyxiation” at higher copolymer concentrations due to cell embedding in the larger multi-chain aggregates. Differently, cells in contact with PEGMA-*ran*-FA at 37 °C, i.e., below *T*_cp_, presented both viability and morphology comparable to those of the control at any given concentration.

These findings show that self-assembling amphiphilic random copolymers of this work are potential candidates as nanocarriers for the effective encapsulation and release of highly hydrophobic drugs whose release rate can be tuned by varying their degree of amphiphilicity, e.g., the length of the oxyethylenic side chain, and/or thermoresponsive behavior, i.e., the cloud point temperature.

## Figures and Tables

**Table 1:** Polymerization data for amphiphilic random copolymers.

Copolymer	Comonomer Feed Ratio (mol/mol) ^1^	Monomers/Initiator Feed Ratio (mol/mol)	Time (h)	Co-Unit Ratioin Copolymer (mol/mol) ^1,2^	*M*_n_^2^ (g/mol)	*M*_n_^3^ (g/mol)	*Đ* ^3^
TEGMA-*ran*-FA	93/7	60/1	26	91/9	16,700	13,200	1.29
PEGMA-*ran*-FA	80/20	50/1	18	80/20	17,800	13,600	1.22

^1^ TEGMA (or PEGMA)/FA. ^2^ By ^1^H NMR. ^3^ By GPC.

**Table 2:** DLS intensity peaks for the synthesized copolymers at a concentration of 30 mg/mL in water, below and above *T*_cp_.

Copolymer	*D*_h_ (Peak 1) (nm)	*D*_h_ (Peak 2) (nm)	*D*_h_ above *T*_cp_ (nm)
TEGMA-*ran*-FA	8 ± 2	120 ± 30	2500 ± 400 ^a^
PEGMA-*ran*-FA	6 ± 1	400 ± 100	600 ± 200 ^b^

^a^ Analysis was carried out at 41 °C. ^b^ Analysis was carried out at 60 °C.

**Table 3:** Encapsulation efficiency (EE) and drug loading (DL) data for Combretastatin A-4 encapsulated in the amphiphilic random copolymers at room temperature.

Copolymer	Copolymer/CA-4 Ratio (*w*/*w*)	EE (%)	DL (%)
TEGMA-*ran*-FA			
	30/1	89 ± 9	3.0 ± 0.3
	17/1	81 ± 7	4.7 ± 0.4
PEGMA-*ran*-FA			
	30/1	86 ± 10	2.9 ± 0.3
	19/1	88 ± 9	4.8 ± 0.7
	15/1	82 ± 14	5.5 ± 0.9
	10/1	82 ± 15	9.0 ± 2.0

## Data Availability

Data is contained within the article.
